# Lactylation Genes LDHA and LDHC Alleviate Osteoarthritis by Reducing Specific B‐Cell Expression: Mechanistic Exploration and Experimental Validation

**DOI:** 10.1111/jcmm.70935

**Published:** 2025-12-03

**Authors:** Jingkai Di, Zijian Guo, Yijing Di, Tingting Chen, Yingda Qin, Xinglong Xing, Chuan Xiang

**Affiliations:** ^1^ Department of Orthopedics Second Hospital of Shanxi Medical University Taiyuan China; ^2^ Shanxi Key Laboratory of Bone and Soft Tissue Injury Repair Taiyuan China; ^3^ The First Hospital of Shanxi Medical University Taiyuan China; ^4^ The Fifth Hospital of Shanxi Medical University Taiyuan China; ^5^ The Second Hospital of Shanxi Medical University Taiyuan China

**Keywords:** immune cells, knee osteoarthritis, lactate dehydrogenase A, lactate dehydrogenase C, lactylation

## Abstract

This study aims to elucidate the correlation between the lactylation‐related genes and the progression of knee osteoarthritis (KOA). Integrating genome‐wide association study data and expression quantitative trait locus data, Mendelian randomisation (MR) and summary‐data‐based MR analyses assessed the correlation between lactylation‐related genes and KOA, validated candidates in chondrocytes by RNA‐seq, western blotting (WB) and quantitative reverse transcriptase PCR (qRT‐PCR). Moreover, CCK‐8, transwell migration and scratch assays were conducted to assess the proliferation/migration. Finally, mediation analysis was utilised to explore the downstream mechanisms. LDHA and LDHC expression was significantly downregulated in the KOA group, as confirmed by RNA‐seq, WB and qRT‐PCR analyses. Functional experiments also confirmed that the down‐regulation of LDHA or LDHC expression could produce a chondrocyte proliferation and migration inhibitory effect similar to that of the OA group, while restoring the expression of these two genes could significantly reverse this phenotype. Furthermore, mediation analysis revealed that CD38 on IgD + CD24− B lymphocytes mediated the protective effect of these genes against KOA, with CD25+ memory B cells also serving as mediators for LDHC's impact on KOA. LDHA and LDHC are identified as therapeutic targets for KOA, providing promising targets and ideas for the development of new treatment strategies.

**Abbreviations**
AARS: Alanyl‐tRNA synthetase
cDNA: complementary DNA
CREBBP: the CREB‐binding protein
DEG: differentially expressed gene
EP300: E1A binding protein p300
eQTL: expression quantitative trait locus
ESCO: establishment of sister chromatid cohesion
FDR: false discovery rate
GEO: Gene Expression Omnibus
GWAS: genome‐wide association study
HDAC: histone deacetylase
HEIDI: heterogeneity in dependent instruments
IV: instrumental variable
IVW: inverse variance weighting
KAT: lysine acetyltransferase
KAT2A: histone acetyltransferase 2A
KOA: knee osteoarthritis
LD: linkage disequilibrium
LDH: lactate dehydrogenase
MAF: minor allele frequency
MAVS: mitochondrial antiviral signalling proteins
MR: Mendelian randomisation
OA: osteoarthritis
PBS: phosphate buffered saline
PTM: post‐translational modification
qRT‐PCR: quantitative reverse transcriptase PCR
SIRT: sirtuin
SLC16A: solute carrier family 16 member A
SMARCA: SWI/SNF‐related, matrix‐associated, actin‐dependent regulator of chromatin, subfamily A
SMR: summary‐data‐based MR
SNP: single nucleotide polymorphism
TSMR: two‐step Mendelian randomisation
UKBB: UK Biobank
WB: Western blotting

## 1 Introduction

Osteoarthritis (OA) is a progressive joint condition mainly distinguished by the deterioration of articular cartilage and diffuse inflammation of the synovium, with middle‐aged and elderly populations being at high risk. As of 2021, over 600 million people worldwide suffer from osteoarthritis, accounting for approximately 7.7% of the global population [1]. The knee joint, as one of the largest weight‐bearing joints in the body, is one of the earliest and most susceptible sites for OA development. Currently, there are more than 595 million patients with knee osteoarthritis (KOA) worldwide, accounting for 7.6% of the global population, which is a 74.9% increase compared to 2020 [2]. Current clinical treatment strategies for KOA mainly focus on alleviating pain and improving joint function, while end‐stage KOA is often associated with lifelong disability, leading patients to require knee joint replacement surgery, which significantly increases their economic burden [3]. An epidemiological study based on the Australian population revealed that healthcare expenditures for OA reached $2.1 billion in 2015 and are expected to exceed $2.9 billion by 2030 [4]. As the burden of KOA disease increases, it becomes particularly important to conduct in‐depth research on the pathogenesis of KOA and to identify new therapeutic targets.

Recently, scientists have increasingly focused on the potential molecular mechanisms involved, with particular attention to epigenetic regulation. Post‐translational modifications (PTMs), such as acetylation, methylation and phosphorylation, are recognised as essential components of epigenetic regulatory mechanisms that may alter the structure and function of proteins in cartilage, thereby affecting key biological processes such as chondrocyte metabolism, signalling and immune response. Lactylation, as an emerging form of epigenetic modification, has gained increasing attention. The lactylation process typically occurs on lysine residues derived from glycolytic lactate, catalysed by the transcriptional coactivator p300 and removed by class I histone deacetylases [5]. To date, lactylation has been shown to be associated with various physiological or pathological processes. For example, lactate dehydrogenase A (LDHA) can induce the lactylation of histones, thereby regulating the transcriptional activity of the TPI1 gene and mediating the progression of OA [6]. Additionally, lactate accumulation upregulates the expression of the ARG2 gene, activating the mTOR/S6K1 pathway and inducing senescence in fibroblast‐like synoviocytes, thus promoting the occurrence of OA [7]. However, the role and potential mechanisms of lactylation in cartilage degradation based on KOA remain relatively unknown. Therefore, clarifying the role of lactylation‐related genes in cartilage degeneration is crucial for further revealing its potential pathological mechanisms and therapeutic targets.

Genome‐wide association studies (GWAS) have discovered numerous genetic variations linked to different complex characteristics through large‐scale analyses, revealing the potential genetic basis of disease phenotypes [8]. Within the human genome, certain single nucleotide polymorphisms (SNPs) that influence gene expression amounts are termed expression quantitative trait loci (eQTLs). The integration of GWAS and eQTL data can uncover potential causal relationships between key genes and complex traits for disease treatment targets [9].

In this study, by integrating the aforementioned data, we identified potential associations between the levels of genes associated with lactylation and the progression of knee osteoarthritis (KOA). We plan to further validate the strongly associated genes through RNA‐seq data from human knee cartilage tissues and experimental approaches. Additionally, the study will explore their downstream mechanisms. Our research aims to elucidate the specific changes in the expression of lactylation‐related genes concerning the progression of KOA, providing new insights into the possible pathological mechanisms and treatment targets for KOA.

## 2 Method

### 2.1 eQTL Data Source

**FIGURE 1:**
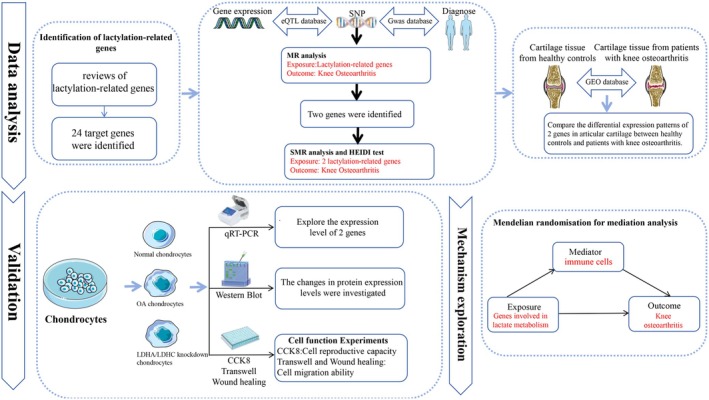
The flow chart of this study.

The overall work flow of this study was shown in Figure 1. To investigate the possible causal correlation between lactylation genes and KOA, we extracted a total of 24 genes associated with lactylation from the reviews [10, 11, 12]. The genetic data for these lactylation‐related genes were obtained from the publicly available expression data of the eQTLGen database, which includes 16,987 eQTL gene data from 31,684 healthy European blood samples [13]. This data is helpful for identifying genetic variations associated with gene expression levels. We specifically focused on cis‐eQTLs with a minor allele frequency (MAF) exceeding 1% and located within 1 Mb of the target gene's genetic variation range.

### 2.2 Outcome Data Source

The summary statistics for KOA were from the UK Biobank (UKBB), which included 31,267 clinically diagnosed KOA cases and 184,763 controls. We downloaded these data from the publicly accessible GWAS catalogue website [14].

### 2.3 Selection of Genetic Instruments

To ensure the robustness of the potential association between lactylation genes and KOA, we performed rigorous quality control procedures to select genetic instrumental variables (IVs) that met three core Mendelian randomisation (MR) assumptions. First, SNPs with significance across the entire genome threshold of less than 5 × 10^−8^ were selected as IVs. Additionally, we conducted linkage disequilibrium (LD) clustering analysis to exclude SNPs that were in strong LD (*r* ^2^ < 0.001, window = 10 Mb). SNPs with a MAF of less than 0.01 were also excluded. Finally, to avoid weak instrument bias for the selected SNPs, we calculated the *F*‐statistic for each independent SNP using *F* = *R* ^2^ × (*N*−2)/(1−*R* ^2^); only high‐intensity SNPs (*F* > 10) were included in the analysis.

### 2.4 Two‐Sample Mendelian Randomisation

Our study used STROBE‐MR guidance to assess the integrity of the entire process (Table S1). Two‐sample MR analysis was utilised to infer the causal association between the levels of genes associated with lactylation and the incidence of KOA. The inverse variance weighted (IVW) method was considered the primary random effects model, supported by sensitivity analyses. Cochran's *Q* was utilised to evaluate potential heterogeneity between the IVW and MR–Egger methods. Additionally, the MR–Egger intercept is an indicator for assessing the directional pleiotropy between IVW and the outcome; a significant deviation from zero would suggest the presence of pleiotropic bias. The MR–PRESSO test addresses potential pleiotropy impacts on causal inference by removing identified outliers. Finally, the leave‐one‐out method was utilised to identify the possible influence of individual SNPs on the results and assess the reliability of the findings. All methods were conducted using the ‘TwoSampleMR’ and ‘MRPRESSO’ software packages, with a *p*‐value less than 0.05 considered statistically significant.

### 2.5 Summary‐Data‐Based Mendelian Randomisation

Summary‐data‐based MR (SMR) analysis was utilised to assess the connections among gene expression levels corresponding to identified protein targets and specific complex biological traits. In this study, the SMR method was employed to verify the potential correlation between the strongly associated lactylation genes and KOA identified in the previous step. Additionally, we utilised the heterogeneity in dependent instruments (HEIDI) test to assess whether the relationship was attributed to shared genetic variation, as opposed to LD. A *p*‐value less than 0.05 was deemed statistically significant; additionally, a HEIDI test *p*‐value greater than 0.05 suggested that the relationship could be attributed to shared genetic variation. Both SMR analysis and HEIDI tests were carried out using SMR software (V.1.03).

### 2.6 RNA‐Seq Data Source

The total RNA‐seq dataset of human knee cartilage was derived from the Gene Expression Omnibus (GEO) database. We selected the OA highly correlated GSE114007 RNA‐seq data, including 20 patients with OA samples and 18 healthy controls of cartilage tissue RNA‐seq data [15]. The samples were taken from the weight‐bearing positions of the medial and lateral condyles of different individuals. Healthy cartilage samples were acquired from 5 women and 13 men having an average age of 38 who had never suffered joint disease or trauma during their lifetime and were processed within 3 days of death to ensure data reliability. In addition, information on gene levels in knee cartilage tissue impacted by OA came from 12 women and 8 men who had undergone knee replacement surgery, with an average age of 66 years. All data is publicly available, so no additional ethical review is required.

### 2.7 Differentially Expressed Genes Analysis

According to the needs of the study, the cartilage tissue samples of KOA patients and the control group were extracted for differentially expressed genes (DEGs) analysis. The false discovery rate (FDR) method was utilised to reduce false positive results due to multiple comparisons, and genes with FDR < 0.05 after correction were considered to be significantly different in expression between the control and KOA groups. We utilised the ‘Limma’ software package to recognise DEGs between KOA patients and healthy controls.

### 2.8 Cell Transfection

To deeply investigate the functions of lactylation‐related genes in the occurrence and progression of OA, we respectively constructed gene knockdown and overexpression models of LDHA and LDHC. 2 × 10^5^ chondrocytes in the logarithmic growth period were inoculated on the six‐well culture plate. When the cell fusion degree reached 70%, Lipofectamine TM 2000 transfection reagent was used. Specific siRNA targeting LDHA and LDHC and its control Public Protein/Plasmid Library (Nanjing, Jiangsu Province, China) were introduced into cells. Subsequently, the transfected cells were placed in an incubator for 8 h to ensure that the siRNA was fully combined with the target gene mRNA, thus achieving the gene knockdown effect and providing conditions for subsequent experimental analysis. Finally, the knockdown effect of LDHA and LDHC genes was detected by quantitative reverse transcriptase PCR (qRT‐PCR) and Western blot (WB).

The gene overexpression model is based on the NCBI reference sequence (LDHA: NM_017266.3; LDHC: NM_017025.2), We cloned the cDNA of LDHA and LDHC into the pcDNA3.1 vector respectively, and constructed the LDHA/pcDNA3.1 and LDHC/pcDNA3.1 overexpression plasmids. Transfection was performed respectively using Lipofectamine TM 3000 transfection reagent (Invitrogen, Thermo FisherScientific). After 48 h, positive clones were screened in a medium containing purinomycin and amplified for culture to obtain stably transfected cell lines. Then additional experiments were performed.

### 2.9 CCK8 Assay

In this experiment, immortalised human cartilage cell line (IM‐H488) was purchased from IMMOCELL. (Xiamen, China) The CCK8 assay was utilised to assess chondrocyte proliferation. Chondrocytes with normal and LDHA/LDHC genes knocked down or overexpressed were inoculated into 96‐well plates with a density of 200 μL/well (1 × 10^4^ cells/well) and incubated at 37°C for 24 h to ensure good adhesion of chondrocytes. In addition, IL‐1β was added to construct a chondrocyte model in the OA group when the cell fusion in the control group reached 80%. After treatment, 10 μL CCK‐8 and 90 μL DMEM were included in every well and maintained for 1 h. Finally, absorbance was recorded at 450 nm utilising the SpectraMax M5 enzyme label reader (Molecular Devices, USA).

### 2.10 Transwell Assay

Transwell assay was utilised to determine the migration ability of chondrocytes after the expression of the control group, OA group and knockdown or overexpressed lactylation gene. 1 × 10^6^ chondrocytes were transferred in 100 μL serum‐free culture medium and subsequently placed into the upper chamber of the 24‐well plate, while 600 μL of complete medium was supplied to the lower chamber to provide growth factors required for chondrocyte migration. After incubation at 37°C for 12 h, the cells in the lower chamber were preserved with 4% paraformaldehyde for 15 min and then stained with 1% crystal violet solution. After binding for a period of time, phosphate‐buffered saline (PBS) was utilised to remove the residual staining solution. The migrated cell photos were photographed by fluorescence microscopy (Leica, Germany) and the number of chondrocytes stained in the lower chamber was counted.

### 2.11 Scratch Assay

The scratch assay was utilised to further evaluate the effect of genes on the lateral migration ability of chondrocytes [16]. Chondrocytes in the logarithmic growth phase were seeded in six‐well plates at a ratio of 1 × 10^6^. After the confluence reaches 90%, use a 200 μL sterile pipette tip to draw a straight line on the cell culture layer, and then wash twice with PBS solution to remove cell debris. Chondrocytes were observed and imaged using a fluorescence microscope (Leica, Germany) at three different time points of 0, 12 and 24 h, and the wound area was evaluated using Image J software.

### 2.12 qRT‐PCR Assay

TRIzol reagents (Invitrogen, Carlsbad, CA) were utilised to rupture chondrocytes in different states and extract total RNA from them. The isolated RNA was subsequently converted into complementary DNA (cDNA) via the Primescript RT kit (Takara, Chiga, Japan). Next, the qRT‐PCR procedure was carried out utilising the SYBR Green RT‐PCR kit (Takara, Chiga, Japan) and the QuantStudio 6 Flex real‐time fluorescence quantitative PCR instrument (Thermo Fisher, USA). The advancement of the amplification process was tracked instantly through the detection of fluorescence intensity. Primer sequences are shown in the attached file (Table S2).

### 2.13 Western Blot

Protein was extracted from cells lysed by cell lysate containing protease inhibitors (AR0102, Boster, Wuhan, China). BCA assay kit (AR1189, Boster, Wuhan, China) was utilised for quantitative determination. After separation by constant pressure and constant current electrophoresis, the target protein was blotted onto the PVDF membrane (Milipore, USA). Next, the membrane is sealed with a sealing solution of 5% skim milk powder. Specific antibody LDHC Rabbit pAb (A15003; 1:1000) and LDHA Rabbit pAb (A1146; 1:2000) were added to the membrane, and all were provided by Abclonal (Wuhan, China) and incubated at 4°C overnight. After three TBST washes, Goat Anti‐rabbit IgG (H + L) (BA1055, Boster, Wuhan, China) was added and incubated at room temperature for 2 h. GAPDH Rabbit mAb (A19056; 1:100,000) and β‐actin rabbit mAb (AC026; 1:100,000) were selected as housekeeping controls for gene knockdown and plasmid‐mediated overexpression experiments, respectively, provided by Abclonal (Wuhan, China). Protein bands were visualised by using FGSuper Sensitive ECL Luminescence Reagent (MA0186, Meilunbio, Dalian, China). ChemiDoc XRS+ Gel Imaging System (BIO‐RAD, California, USA) was utilised to detect protein expression levels. Each experiment was repeated three times. In addition, the ratio of grey intensities of target bands compared to internal reference bands can be utilised to calculate the relative expression level of proteins.

### 2.14 Mediation Analysis

In order to explore the downstream mechanisms that link lactate genes to KOA, a two‐step Mendelian randomisation (TSMR) analysis was applied to evaluate the direct and indirect association of lactate genes to KOA. TSMR assumes no interaction between exposure and mediators, while attenuating the bias of high LD associations between SNPs. First, the causal effects of lactylation‐related genes on immune cells (β1) were calculated separately, and then the effects of these mediators on KOA (β2) were estimated. The analysis evaluated the associated effects of each step separately and explored how lactate genes influence the onset of KOA through mediating variables. The formula ‘β1_2 = β1*β2’ was utilised to estimate the mediated proportion of KOA affected by mediating factors, and the standard error of indirect effects was obtained via the delta approach.

## 3 Result

### 3.1 Recognition of Potential Genes for KOA

**FIGURE 2:**
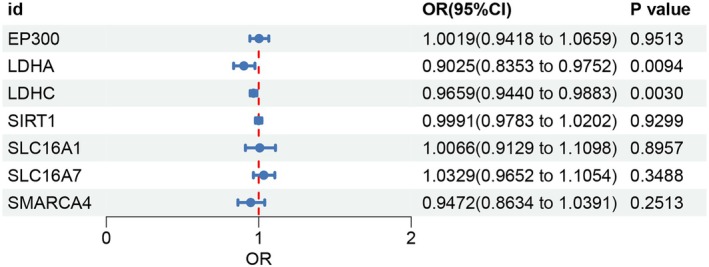
Forest plot of the relationship between lactylation‐related genes and KOA based on MR analysis. KOA, knee osteoarthritis; MR, Mendelian randomisation.

According to the reviews, 24 lactylation‐related genes were included in the study, including EP300, CREBBP, KAT2A, KAT5, KAT8, KAT7, ESCO2, AARS1, AARS2, HDAC1, HDAC2, HDAC3, HDAC8, SIRT1, SIRT2, SIRT3, SIRT6, SMARCA4, LDHA, LDHB, LDHC, SLC16A4, SLC16A1, SLC16A7. The results showed that LDHA and LDHC genes may be negatively related to the hazard of KOA (LDHA: OR = 0.903, 95% CI = 0.835–0.975, *p* < 0.05; LDHC: OR = 0.966, 95% CI = 0.944–0.988, *p* < 0.05) (Figure 2). In sensitivity analysis, no heterogeneity (*p* > 0.05) or horizontal pleiotropy (*p* > 0.05) was found in LDHA and LDHC.

### 3.2 SMR Analysis and HEIDI Test

In order to further explore whether the influence of the above two genes on KOA is related to the expression level of genes, we conducted SMR analysis and HEIDI detection. We found that the expression levels of LDHA (OR = 0.899, 95% CI = 0.815–0.991, *p* < 0.05) and LDHC (OR = 0.967, 95% CI = 0.936–0.998, *p* < 0.05) reduced the hazard of KOA. The results of the HEIDI test showed that the SNPs of the two genes did not have pleiotropy (p‐HEIDI > 0.05) (Table 1).

**TABLE 1:** The SMR and HEIDI tests show the association of LDHA and LDHC with KOA.

Gene	Probe ID	topSNP	SMR association			OR (95% CI)	HEIDI test	
			*b*	SE	*p*		*p*	nSNPs
LDHA	ENSG00000134333	rs6486423	−0.106762	0.0498941	0.0324	0.899 (0.815–0.991)	0.226	20
LDHC	ENSG00000166796	rs78454952	−0.0340216	0.0164169	0.0382	0.967 (0.936–0.998)	0.338	20

Abbreviations: *b*, beta; *p*, *p* value; SE, standard error.

### 3.3 Differential Expression Analysis Based on RNA‐Seq

**FIGURE 3:**
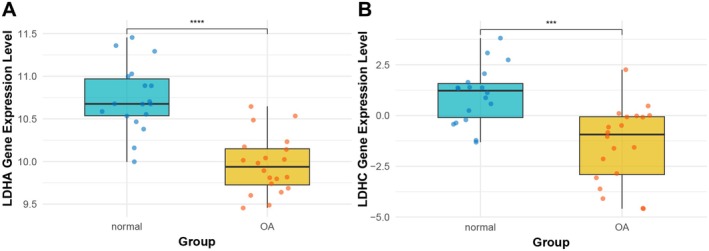
LDHA (A) and LDHC (B) expression in normal and KOA cartilage tissue retrieved from GEO datasets. GEO, Gene Expression Omnibus; KOA, knee osteoarthritis; LDHA, lactate dehydrogenase A; LDHC, lactate dehydrogenase C. ***p < 0.001, ****p < 0.0001.

RNA‐seq data from cartilage of 18 healthy controls and 20 KOA patients from the GSE114007 RNA‐seq dataset in the GEO database were applied to compare the expression differences of LDHA and LDHC lactylation‐related genes between the two groups. The results suggested that the expression of both genes in KOA patients' cartilage was decreased compared with normal cartilage tissue (LDHA: *p* < 0.0001, LDHC: *p* < 0.001) (Figure 3).

### 3.4 Validation of the Genetic Expression of LDHA and LDHC

**FIGURE 4:**
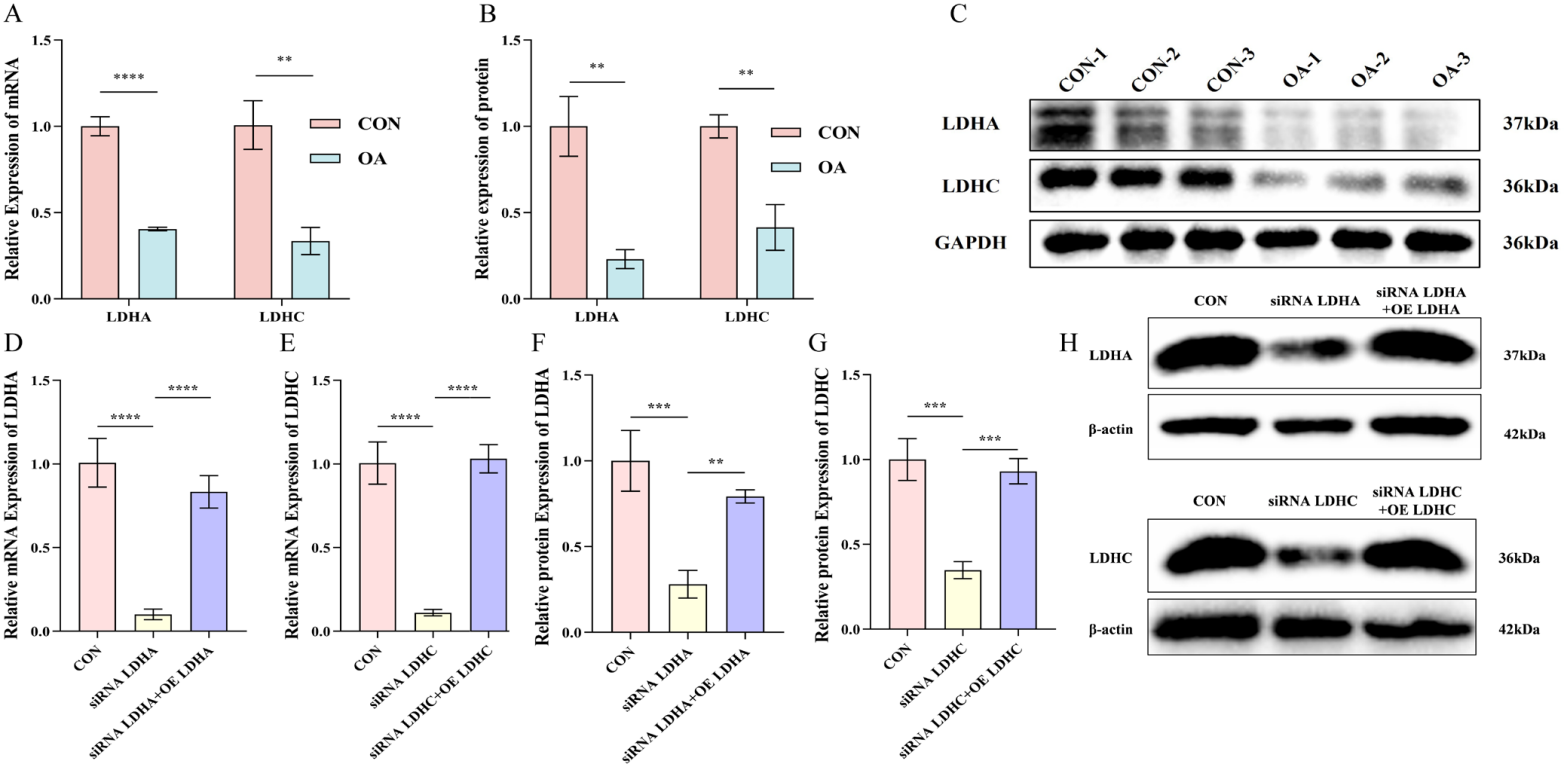
Immortalized human chondrocyte cell lines for evaluating the expression of LDHA and LDHC. (A) qRT‐PCR for mRNA expression of LDHA and LDHC in OA and normal chondrocytes. (B, C) WB analysis of LDHA and LDHC protein expression in chondrocytes from OA and control groups, each experiment was repeated three times. (D, E) Knockdown and overexpression efficiency of the target gene validated at the mRNA level by qRT‐PCR. (F, G and H) WB validation of target gene knockdown and overexpression at the protein level. GAPDH, glyceraldehyde‐3‐phosphate dehydrogenase; LDHA, lactate dehydrogenase A; LDHC, lactate dehydrogenase C; OA, osteoarthritis; qRT‐PCR, quantitative reverse transcriptase PCR; WB, western blot (**p < 0.01, ***p < 0.001, ****p < 0.0001).

According to the results of qRT‐PCR (LDHA: *p* < 0.0001; LDHC: *p* < 0.01; Figure 4A) and WB (*p* < 0.01; Figure 4B,C), the expression levels of LDHA and LDHC in chondrocytes of the KOA group were significantly reduced.

To further clarify the biological functions of LDHA and LDHC in KOA, in this study, chondrocyte models of LDHA and LDHC gene knockdown were successfully constructed first by siRNA transfection technology, and their knockdown efficiency was verified by qRT‐PCR (*p* < 0.0001) and WB (*p* < 0.001). To further ensure the reliability of the results and eliminate potential off‐target effects, LDHA and LDHC overexpression plasmids were constructed to achieve stable overexpression in chondrocytes, respectively. Their expression efficiency was once again verified at the mRNA (*p* < 0.0001; Figure 4D,E) and protein (LDHA: *p* < 0.01; LDHC: p < 0.001; Figure 4F–H) levels.

### 3.5 Validation of the Genetic Function of LDHA and LDHC

**FIGURE 5:**
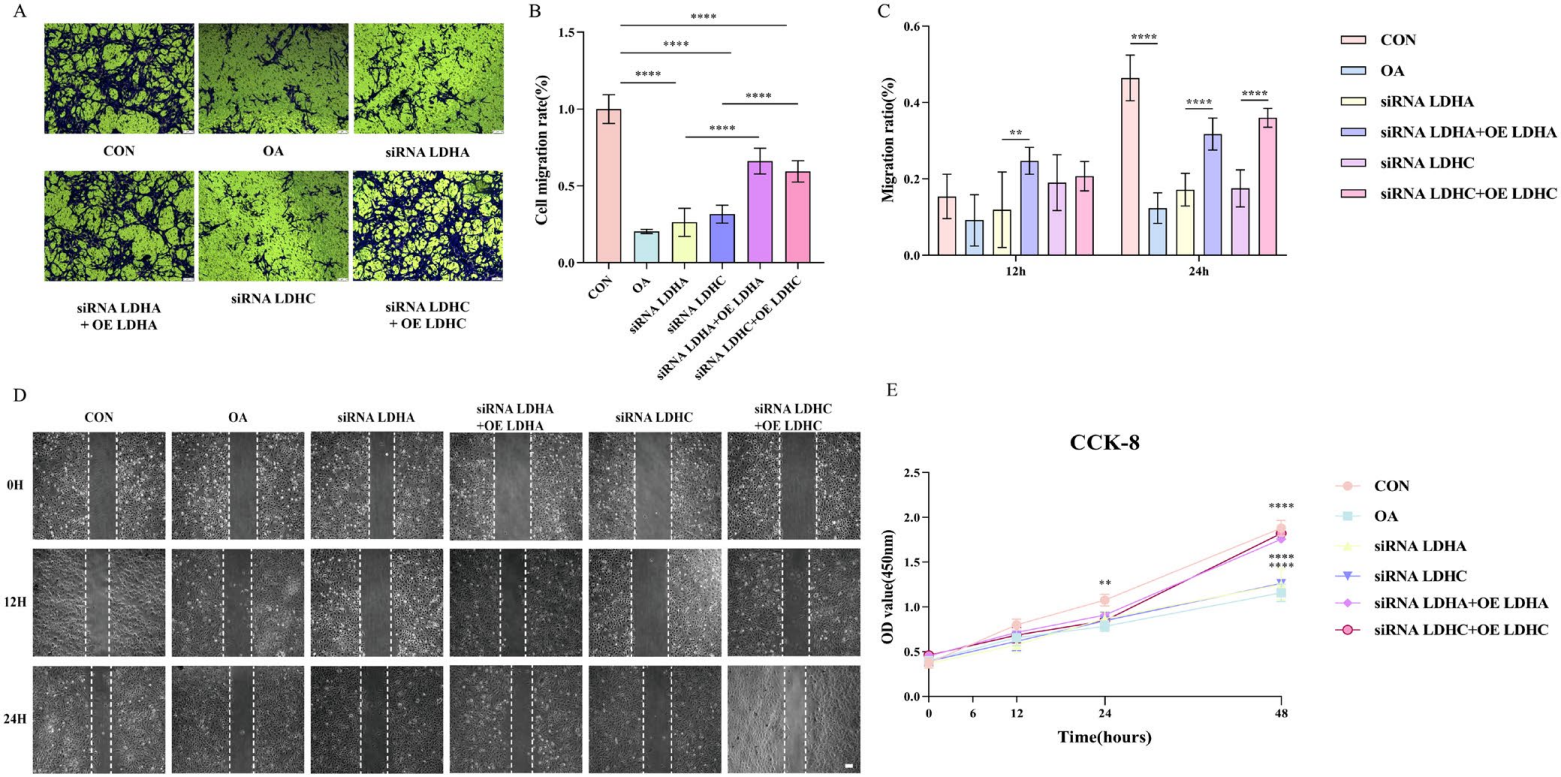
Immortalized human chondrocyte cell lines for evaluating the function of LDHA and LDHC. (A, B) Transwell migration assay evaluating chondrocyte migration capacity under LDHA/LDHC genetic modulation (knockdown/overexpression) and OA conditions. (C, D) Scratch assay evaluating chondrocyte migration capacity under LDHA/LDHC genetic modulation (knockdown/overexpression) and OA conditions. (E) CCK‐8 assay evaluating chondrocyte migration capacity under LDHA/LDHC genetic modulation (knockdown/overexpression) and OA conditions. OA, osteoarthritis (**p < 0.01, ****p < 0.0001).

Subsequently, functional experiments were conducted to evaluate the effects of LDHA and LDHC on the proliferation and migration abilities of chondrocytes. The transwell migration assay (Figure 5A,B) and the scratch healing assay (Figure 5C,D) revealed that the knockdown of LDHA/LDHC significantly weakened the migration ability of chondrocytes to a degree similar to that of the IL‐1β‐induced OA group (*p* < 0.0001). It is worth noting that gene re‐overexpression can effectively salvage the chondrocyte migration function defect caused by siRNA interference (*p* < 0.0001). Furthermore, the CCK‐8 experiment demonstrated that IL‐1β treatment significantly inhibited chondrocyte proliferation (*p* < 0.0001). The knockdown of LDHA or LDHC could also produce a similar proliferation inhibitory effect to the OA group (*p* < 0.0001), while restoring the expression of these two genes could significantly reverse this phenotype (*p* < 0.0001; Figure 5E).

### 3.6 Mediation Analysis

It has been shown that the process of lactylation can affect the activity of immune cells. The incidence of OA is highly influenced by immune cells, and the elevation of immune cells can be found in the articular cartilage affected by OA. Therefore, we hypothesised that lactylation‐related genes may be mediated by immune cells to exert their influence on KOA. Mediation analysis showed that the effects of both genes on KOA were mediated by B lymphocytes of CD38 on IgD + CD24−. The mediating effects of CD38 on IgD + CD24− B lymphocytes on LDHA were −0.020 (95% CI = −0.068 to 0.029). The mediated proportion was 19%; the mediating effect on LDHC was −0.020 (95% CI = −0.068 to 0.029), explaining 56.3% of the mediation. In addition, LDHC can mediate through CD25+ memory B cells with a mediating effect of −0.005 (95% CI = −0.052 to 0.041) and a mediating ratio of 15.7% (Table 2).

**TABLE 2:** Mediating effect of B lymphocytes on the risk of LDHA/LDHC‐KOA.

Mediator	Exposure	Total effect^a^	Direct effect^b^	Mediation effect^c^	Mediated proportion
		Effect size (95% CI)	Effect size	Effect size (95% CI)	(%)
B cell (CD38 on IgD + CD24−)	LDHA	−0.103 (−0.180 to −0.025)	−0.083	−0.020 (−0.068 to 0.029)	19.0%
	LDHC	−0.035 (−0.058 to −0.012)	−0.015	−0.020 (−0.068 to 0.029)	56.3%
B cell (CD25 on memory B cell)	LDHC	−0.035 (−0.058 to −0.012)	−0.029	−0.005 (−0.052 to 0.041)	15.7%

^a^ Total effect: the effect of exposure on the knee osteoarthritis.

^b^ Direct effect: the effect of exposure on the knee osteoarthritis, not explained by the mediator.

^c^ Mediation effect: the effect of exposure on the knee osteoarthritis acting through the mediator.

## 4 Discussion

KOA is a degenerative joint condition with a high incidence among people of middle and advanced age, and its high incidence and disability rate seriously reduce the health status of patients. Currently, clinical drugs targeting KOA mainly focus on alleviating symptoms, and there is an urgent need to open up new intervention pathways targeting its pathological mechanism [17].

Through MR and SMR analysis, our study found that the expressions of LDHA and LDHC, which are related to lactic acid, were significantly down‐regulated in the KOA group, suggesting a potential causal relationship between the two and the development of KOA. Further, GEO data and related experiments also verified the above conclusions. Functional experiments also confirmed that the down‐regulation of LDHA or LDHC expression could produce a chondrocyte proliferation and migration inhibitory effect similar to that of the OA group, while restoring the expression of these two genes could significantly reverse this phenotype. In addition, we delved into the downstream mechanisms of the effects of LDHA and LDHC on KOA; specifically, CD38 on IgD + CD24− B lymphocytes mediates the common pathway of the protective effects of the above two genes on KOA, and CD25+ memory B cells are also mediators of the effect of LDHC on KOA risk. These findings revealed the key role of lactate metabolism and immune regulation in KOA, providing potential targets for developing novel therapeutic strategies for KOA.

The LDHA and LDHC genes encode two different subtypes of lactate dehydrogenase (LDH), which predominantly catalyse the transformation of pyruvate to lactic acid. Lactic acid is the end product of glycolysis, and in addition to being a metabolic fuel for cells, it is also closely related to physiological or pathological procedures such as redox homeostasis, immune response and inflammation [18, 19]. These processes are considered to be one of the central links in the occurrence and progression of KOA [20, 21, 22]. In the pathological process of KOA, oxidative stress is one of the important factors leading to chondrocyte injury [23]. Lactate dehydrogenase can maintain the homeostasis of intracellular redox reactions by regulating the ratio of NADH/NAD+, thereby reducing oxidative stress and preventing apoptosis of chondrocytes [24]. The ability of LDHA and LDHC genes encoding lactate dehydrogenase to mitigate oxidative damage may be the key to their protective effect. In addition, lactic acid, as a metabolite of lactate dehydrogenase, can not only inhibit the expression of cartilage matrix degradation genes in OA chondrocytes, but also promote matrix synthesis [25]. When the expression of LDHA and LDHC genes is reduced, it will lead to the decrease of lactic acid levels, weaken the inhibition of cartilage matrix degradation genes, and further lead to the degeneration of knee articular cartilage. In addition to the metabolic action itself, lactic acid may also act as a substrate for histone lactylation, which can affect the progression of KOA [5, 26]. A study exploring the metabolic regulation of gene expression by histone lactylation showed that lactic acid exhibited a protective effect on bone and joint during KOA progression, and it could activate gene expression in M2‐like macrophages through histone lysine lactylation, thereby exerting anti‐inflammatory properties. Persistent low‐grade inflammation is often seen as a significant hallmark of KOA. Thus, the study of lactylation‐related genes opens up new research perspectives on the potential pathologic mechanisms of KOA and its therapeutic targets.

Notably, by deeply examining the downstream mechanisms through which lactate gene expression steers the pathogenesis of KOA, we confirmed that the LDHA and LDHC genes could significantly antagonise the promoting effect of IgD+ CD24− on CD38 B lymphocytes on the occurrence and progression risk of KOA. In addition, CD25+ memory B cells are also mediators of the effect of LDHC on KOA risk.

The development and maturation of B cells are accompanied by changes in various surface molecules. Specifically, the expression of CD24 is high in the early stage of B cells, but with the maturity and activation of B cells, especially after antigenic stimulation, the expression of the surface molecule CD24 is significantly decreased, while the content of immunoglobulin IgD is relatively increased, which jointly marks the mature state of B cells. Notably, the expression of CD38 and CD25 is significantly increased after B cell activation, which not only reflects the maturity and activation state of B cells, but also marks the transformation of B cells into plasma cells and the improvement of antibody production ability [27, 28].

The latest research indicates that lactylation‐related genes can significantly interfere with the normal differentiation of B cells through pathways such as signal pathway interference or metabolite regulation. For instance, RelA was believed to have an activating effect on the transcriptional process during B cell development. However, the new discovery made by Zhao et al. revealed that the key subunit RelA of the NF‐κB pathway underwent lactylation modification at the K310 site, and its DNA binding ability was significantly weakened [29]. Given this, we speculate that when the expression of LDHA and LDHC genes is upregulated, the transcriptional activity of RelA is selectively inhibited, thereby down‐regulating the expression of key differentiation genes such as PAX5 and EBF1, which leads to the blockage of the terminal maturation of B cells. In addition, IFN‐β has been found to increase the absolute number of mature B cells in peripheral blood. Lactic acid, as a signalling molecule of the functional domain of mitochondrial antiviral signalling proteins (MAVS), can prevent MAVS oligomerization and interrupt the RLR signalling cascade reaction, significantly inhibiting the production of IFN‐β [30, 31]. Therefore, LDHA and LDHC may inhibit the differentiation and maturation of B cells by up‐regulating lactic acid levels. It is worth noting that research on the specific molecular mechanisms by which lactylation‐related genes regulate B cell function is still in its infancy, and the precise regulatory network and downstream effector molecules remain to be further analysed.

So far, CD38 is gradually being confirmed as one of the prospective therapeutic targets for the pathogenesis of KOA. For example, in a study of expression differences between KOA patients and healthy controls by Paulo Gil Alabarse et al., it was observed that CD38 expression was significantly upregulated in the patients' chondrocytes, and this conclusion was also validated in mice with cartilage degradation and synovial inflammation [32]. These pathological processes are often considered to be one of the central links in the occurrence and progression of KOA. In addition, an immunohistochemical analysis suggests that CD38‐positive plasma cells may be the best differentiating and diagnostic marker for early onset of arthritis [33]. Specifically, CD38 induces the release and aggregation of ROS in vivo by up‐regulating the expression of angiotensin II [34]. Oxidative stress is one of the important factors leading to the injury of chondrocytes. The imbalance of ROS production and consumption may lead to the damage of key proteins and nucleic acids, thus inducing the storm of apoptosis of chondrocytes and promoting the occurrence and progression of KOA [23]. Further, Seon‐Young Kim et al. confirmed through animal experiments that CD38 can also stimulate angiotensin II‐mediated calcium ion release and promote the process of tissue fibrosis [35]. This fibrosis can aggravate the wear of the synovial and subchondral bones in the knee joints, which is also considered to be one of the main pathological manifestations of KOA disease.

In addition to CD38 on IgD + CD24− B lymphocytes mediating the protective effects of LDHA and LDHC on KOA, our study also found that CD25+ memory B cells are also mediators of the effect of LDHC on KOA risk. In a study using activated B cells co‐cultured with fibroblasts from OA patients, the two co‐induced joint hyperplasia and diffuse inflammation. Notably, TNF‐α and IL‐1β, produced and released by activated CD25 + B cells, have been shown to be key to the release of cartilage‐damaging inflammatory factors [36]. In addition, the possible induction of local inflammation by activated CD25 + B cells was also confirmed in a study exploring the correlation between periodontitis and immune cells, which observed that CD25 + B cells can produce the local inflammatory factor IL‐35 [37]. IL‐35 accumulates in large quantities in chondrocytes, thereby enhancing the induction of apoptosis of osteoclasts by TNF‐α [38]. The imbalance of osteoclasts may interfere with the remodelling process of subchondral bone, resulting in enhanced bone transformation and deterioration of bone trabeculae, thus aggravating osteoporosis and cartilage injury around the joint [39].

Our research has many advantages. Combining GEO databases and MR methods, the study for the maiden time validated a potential correlation between the lactylation‐related genes LDHA and LDHC and KOA, underscoring their potential as actionable prevention and therapeutic targets for KOA. At the same time, strict screening conditions were applied to ensure that bias in the results was minimised. Secondly, we further confirmed the reliability and accuracy of lactylation‐related genes mediating the pathogenesis of KOA through gene expression and functional experiments, respectively. However, there are some shortcomings in our current study. First, GWAS data on disease outcomes are derived primarily from genetic variation in populations of European descent, which means that the results are skewed in other ethnic groups. Moreover, due to current experimental limitations, this study has not yet fully elucidated the specific downstream target gene networks regulated by LDHA/LDHC. This mechanistic gap warrants further investigation using gene‐editing technologies combined with transcriptomic and metabolomic analyses. In addition, our experiments are limited to the cellular level, which to some extent limits the extrapolation of the findings in animal models. Therefore, in future studies, we plan to introduce corresponding animal experiments to more fully validate our findings.

## 5 Conclusion

Overall, lactylation‐related genes LDHA and LDHC have been identified as potential therapeutic targets for KOA, and both are closely related to the proliferation and migration capabilities of chondrocytes. Notably, CD38 on IgD + CD24− B lymphocytes mediate the common pathway through which these two genes provide protection against KOA. In addition, CD25+ memory B cells are also mediators of the effect of LDHC on KOA risk. These findings reveal the key roles of lactate metabolism and immune regulation in KOA, providing potential targets and innovative ideas for the development of new therapeutic strategies.

## Author Contributions

**Jingkai Di:** conceptualization (equal), methodology (equal), writing – review and editing (equal). **Zijian Guo:** formal analysis (equal), investigation (equal), writing – review and editing (equal). **Yijing Di:** visualization (equal), writing – original draft (equal). **Tingting Chen:** writing – original draft (equal). **Yingda Qin:** writing – original draft (equal). **Xinglong Xing:** writing – original draft (equal). **Chuan Xiang:** funding acquisition (equal), writing – review and editing (equal).

## Ethics Statement

The authors have nothing to report.

## Consent

The authors have nothing to report.

## Conflicts of Interest

The authors declare no conflicts of interest.

## Supporting information


**Table S1:** STROBE‐MR checklist of recommended items to address in reports of Mendelian randomisation studies.

**Table S2:** Primer sequences of target genes and GAPDH.

## Data Availability

The data that support the findings of this study are openly available in ‘eQTLGen Consortium’ at https://doi.org/10.1038/s41588‐021‐00913‐z, ‘UK Biobank’ at https://doi.org/10.1097/j.pain.00000000000002922 and ‘GEO DataSets’ at https://doi.org/10.1016/j.joca.2018.07.012.

## References

[1] X. Xie , K. Zhang , Y. Li , et al., “Global, Regional, and National Burden of Osteoarthritis From 1990 to 2021 and Projections to 2035: A Cross‐Sectional Study for the Global Burden of Disease Study 2021,” PLoS One 20, no. 5 (2025): e0324296. 40424273 10.1371/journal.pone.0324296 PMC12111611

[2] GBD 2021 Osteoarthritis Collaborators , “Global, Regional, and National Burden of Osteoarthritis, 1990–2020 and Projections to 2050: A Systematic Analysis for the Global Burden of Disease Study 2021,” Lancet Rheumatology 5, no. 9 (2023): e508–e522. 37675071 10.1016/S2665-9913(23)00163-7 PMC10477960

[3] S. Youn , J. H. Choi , C. Kim , S. M. Kim , and W. S. Choi , “Efficacy and Safety of Diacerein and Celecoxib Combination Therapy for Knee Osteoarthritis: A Double‐Blind, Randomized, Placebo‐Controlled Prospective Study,” Medicine (Baltimore) 102, no. 39 (2023): e35317. 37773836 10.1097/MD.0000000000035317 PMC10545013

[4] I. N. Ackerman , C. Pratt , A. Gorelik , and D. Liew , “Projected Burden of Osteoarthritis and Rheumatoid Arthritis in Australia: A Population‐Level Analysis,” Arthritis Care & Research 70, no. 6 (2018): 877–883. 28898565 10.1002/acr.23414

[5] D. Zhang , Z. Tang , H. Huang , et al., “Metabolic Regulation of Gene Expression by Histone Lactylation,” Nature 574, no. 7779 (2019): 575–580. 31645732 10.1038/s41586-019-1678-1 PMC6818755

[6] J. Xia , Z. Qiao , X. Hao , and Y. Zhang , “LDHA‐Induced Histone Lactylation Mediates the Development of Osteoarthritis Through Regulating the Transcription Activity of TPI1 Gene,” Autoimmunity 57, no. 1 (2024): 2384889. 39086231 10.1080/08916934.2024.2384889

[7] Y. Huang , S. Yue , Z. Yan , et al., “Lactate‐Upregulated ARG2 Expression Induces Cellular Senescence in Fibroblast‐Like Synoviocytes of Osteoarthritis via Activating the mTOR/S6K1 Signaling Pathway,” International Immunopharmacology 142, no. Pt A (2024): 113071. 39236462 10.1016/j.intimp.2024.113071

[8] E. Mountjoy , E. M. Schmidt , M. Carmona , et al., “An Open Approach to Systematically Prioritize Causal Variants and Genes at All Published Human GWAS Trait‐Associated Loci,” Nature Genetics 53, no. 11 (2021): 1527–1533. 34711957 10.1038/s41588-021-00945-5 PMC7611956

[9] P. Kreitmaier , G. Katsoula , and E. Zeggini , “Insights From Multi‐Omics Integration in Complex Disease Primary Tissues,” Trends in Genetics 39, no. 1 (2023): 46–58. 36137835 10.1016/j.tig.2022.08.005

[10] C. Bao , Q. Ma , X. Ying , et al., “Histone Lactylation in Macrophage Biology and Disease: From Plasticity Regulation to Therapeutic Implications,” eBioMedicine 111 (2025): 105502. 39662177 10.1016/j.ebiom.2024.105502 PMC11697715

[11] Y. Wang , P. Li , Y. Xu , et al., “Lactate Metabolism and Histone Lactylation in the Central Nervous System Disorders: Impacts and Molecular Mechanisms,” Journal of Neuroinflammation 21, no. 1 (2024): 308. 39609834 10.1186/s12974-024-03303-4 PMC11605911

[12] W. Yao , X. Hu , and X. Wang , “Crossing Epigenetic Frontiers: The Intersection of Novel Histone Modifications and Diseases,” Signal Transduction and Targeted Therapy 9, no. 1 (2024): 232. 39278916 10.1038/s41392-024-01918-w PMC11403012

[13] U. Võsa , “Large‐Scale Cis‐ and Trans‐eQTL Analyses Identify Thousands of Genetic Loci and Polygenic Scores that Regulate Blood Gene Expression; The eQTLGen Consortium,” Nature Genetics 53 (2021): 1300–1310, 10.1038/s41588-021-00913-z. 34475573 PMC8432599

[14] K. Zorina‐Lichtenwalter , “Genetic Risk Shared Across 24 Chronic Pain Conditions: Identification and Characterization With Genomic Structural Equation Modeling,” Pain 164 (2023): 2239–2252, 10.1097/j.pain.0000000000002922. 37219871 PMC10524350

[15] K. M. Fisch , “Identification of Transcription Factors Responsible for Dysregulated Networks in Human Osteoarthritis Cartilage by Global Gene Expression Analysis,” Osteoarthritis and Cartilage 26 (2018): 1531–1538, 10.1016/j.joca.2018.07.012. 30081074 PMC6245598

[16] F. Yang , S. Duan , J. Liu , et al., “Antitumor Effects of Cannabidiol (CBD) on Osteosarcoma by Targeting TNF‐α/NF‐κB/CCL5 Signaling Axis,” Phytomedicine 145 (2025): 157066. 40680332 10.1016/j.phymed.2025.157066

[17] M. Langworthy , V. Dasa , and A. I. Spitzer , “Knee Osteoarthritis: Disease Burden, Available Treatments, and Emerging Options,” Therapeutic Advances in Musculoskeletal Disease 16 (2024): 1759720X241273009. 10.1177/1759720X241273009 PMC11406648 39290780

[18] X. Lv , Y. Lv , and X. Dai , “Lactate, Histone Lactylation and Cancer Hallmarks,” Expert Reviews in Molecular Medicine 25 (2023): e7. 36621008 10.1017/erm.2022.42

[19] Q. Feng , Z. Liu , X. Yu , et al., “Lactate Increases Stemness of CD8+ T Cells to Augment Anti‐Tumor Immunity,” Nature Communications 13, no. 1 (2022): 4981. 10.1038/s41467-022-32521-8 PMC9448806 36068198

[20] Y. Wei , H. Qian , X. Zhang , et al., “Progress in Multi‐Omics Studies of Osteoarthritis,” Biomarker Research 13, no. 1 (2025): 26. 39934890 10.1186/s40364-025-00732-y PMC11817798

[21] A. Haseeb and T. M. Haqqi , “Immunopathogenesis of Osteoarthritis,” Clinical Immunology 146, no. 3 (2013): 185–196. 23360836 10.1016/j.clim.2012.12.011 PMC4015466

[22] J. A. Bolduc , J. A. Collins , and R. F. Loeser , “Reactive Oxygen Species, Aging and Articular Cartilage Homeostasis,” Free Radical Biology & Medicine 132 (2019): 73–82. 30176344 10.1016/j.freeradbiomed.2018.08.038 PMC6342625

[23] B. Chen , Q. He , C. Chen , et al., “Combination of Curcumin and Catalase Protects Against Chondrocyte Injury and Knee Osteoarthritis Progression by Suppressing Oxidative Stress,” Biomedicine & Pharmacotherapy 168 (2023): 115751. 37879214 10.1016/j.biopha.2023.115751

[24] K. Yudoh , N. vT , H. Nakamura , K. Hongo‐Masuko , T. Kato , and K. Nishioka , “Potential Involvement of Oxidative Stress in Cartilage Senescence and Development of Osteoarthritis: Oxidative Stress Induces Chondrocyte Telomere Instability and Downregulation of Chondrocyte Function,” Arthritis Research & Therapy 7, no. 2 (2005): R380–R391. 15743486 10.1186/ar1499 PMC1065334

[25] X. Zhang , Y. Wu , Z. Pan , et al., “The Effects of Lactate and Acid on Articular Chondrocytes Function: Implications for Polymeric Cartilage Scaffold Design,” Acta Biomaterialia 42 (2016): 329–340. 27345139 10.1016/j.actbio.2016.06.029

[26] Q. Xin , H. Wang , Q. Li , et al., “Lactylation: A Passing Fad or the Future of Posttranslational Modification,” Inflammation 45, no. 4 (2022): 1419–1429. 35224683 10.1007/s10753-022-01637-w PMC9197907

[27] R. R. Hardy and K. Hayakawa , “B Cell Development Pathways,” Annual Review of Immunology 19 (2001): 595–621. 10.1146/annurev.immunol.19.1.595 11244048

[28] P. D. Burrows and M. D. Cooper , “B Cell Development and Differentiation,” Current Opinion in Immunology 9, no. 2 (1997): 239–244. 9099791 10.1016/s0952-7915(97)80142-2

[29] Q. Zhao , Q. Wang , Q. Yao , et al., “Nonenzymatic Lysine D‐Lactylation Induced by Glyoxalase II Substrate SLG Dampens Inflammatory Immune Responses,” Cell Research 35, no. 2 (2025): 97–116. 39757301 10.1038/s41422-024-01060-w PMC11770101

[30] R. D. Schubert , Y. Hu , G. Kumar , et al., “IFN‐β Treatment Requires B Cells for Efficacy in Neuroautoimmunity,” Journal of Immunology 194, no. 5 (2015): 2110–2116. 10.4049/jimmunol.1402029 PMC4340715 25646307

[31] W. Zhang , G. Wang , Z. G. Xu , et al., “Lactate Is a Natural Suppressor of RLR Signaling by Targeting MAVS,” Cell 178, no. 1 (2019): 176–189.e15. 31155231 10.1016/j.cell.2019.05.003 PMC6625351

[32] P. Gil Alabarse , L. Y. Chen , P. Oliveira , H. Qin , and R. Liu‐Bryan , “Targeting CD38 to Suppress Osteoarthritis Development and Associated Pain After Joint Injury in Mice,” Arthritis & Rhematology 75, no. 3 (2023): 364–374. 10.1002/art.42351 PMC9998345 36103412

[33] M. C. Kraan , J. J. Haringman , W. J. Post , J. Versendaal , F. C. Breedveld , and P. P. Tak , “Immunohistological Analysis of Synovial Tissue for Differential Diagnosis in Early Arthritis,” Rheumatology (Oxford, England) 38, no. 11 (1999): 1074–1080. 10556258 10.1093/rheumatology/38.11.1074

[34] A. L. Horenstein , A. C. Faini , and F. Malavasi , “CD38 in the Age of COVID‐19: A Medical Perspective,” Physiological Reviews 101, no. 4 (2021): 1457–1486. 33787351 10.1152/physrev.00046.2020 PMC8313238

[35] S. Y. Kim , B. H. Cho , and U. H. Kim , “CD38‐Mediated Ca^2+^ Signaling Contributes to Angiotensin II‐Induced Activation of Hepatic Stellate Cells: Attenuation of Hepatic Fibrosis by CD38 Ablation,” Journal of Biological Chemistry 285, no. 1 (2010): 576–582. 19910464 10.1074/jbc.M109.076216 PMC2804206

[36] H. Störch , B. Zimmermann , B. Resch , et al., “Activated Human B Cells Induce Inflammatory Fibroblasts With Cartilage‐Destructive Properties and Become Functionally Suppressed in Return,” Annals of the Rheumatic Diseases 75, no. 5 (2016): 924–932. 25985971 10.1136/annrheumdis-2014-206965

[37] Y. Han , C. Yu , Y. Yu , and L. Bi , “CD25+ B Cells Produced IL‐35 and Alleviated Local Inflammation During Experimental Periodontitis,” Oral Diseases 28, no. 8 (2022): 2248–2257. 34129722 10.1111/odi.13939

[38] M. Peng , Y. Wang , L. Qiang , et al., “Interleukin‐35 Inhibits TNF‐α‐Induced Osteoclastogenesis and Promotes Apoptosis via Shifting the Activation From TNF Receptor‐Associated Death Domain (TRADD)‐TRAF2 to TRADD‐Fas‐Associated Death Domain by JAK1/STAT1,” Frontiers in Immunology 9 (2018): 1417. 30061878 10.3389/fimmu.2018.01417 PMC6054960

[39] S. Stegen and G. Carmeliet , “Metabolic Regulation of Skeletal Cell Fate and Function,” Nature Reviews. Endocrinology 20, no. 7 (2024): 399–413. 10.1038/s41574-024-00969-x 38499689

