# Supplementary material for: Lactylation Genes LDHA and LDHC Alleviate Osteoarthritis by Reducing Specific B‐Cell Expression: Mechanistic Exploration and Experimental Validation
Source: J Cell Mol Med. 2025 Dec 3;29(23):e70935. doi: 10.1111/jcmm.70935 (PMC12674964; doi:10.1111/jcmm.70935)
Supplement: Supplementary file 2 — Table S2: Primer sequences of target genes and GAPDH. [file JCMM-29-e70935-s001.docx]

**Table S2** Primer Sequences of Target Genes and GAPDH.

| Gene | Primer |
| --- | --- |
| LDHA-F | 5ʹ-TTGACCTACGTGGCTTGGAAG-3ʹ |
| LDHA-R | 5ʹ-GGTAACGGAATCGGGCTGAAT-3ʹ |
| LDHC-F | 5ʹ-TCATTCCTGCCATAGTCCA-3ʹ |
| LDHC-R | 5ʹ-CAATTACACGAGTTACAGGTA-3ʹ |
| GAPDH-F | 5ʹ-GACATGCCGCCTGGAGAAAC-3ʹ |
| GAPDH-R | 5ʹ-AGCCCAGGATGCCCTTTAGT-3ʹ |

LDHA, lactate dehydrogenase A; LDHC, lactate dehydrogenase C; GAPDH, Glyceraldehyde-3-phosphate dehydrogenase.
